# Supplementary material for: uPAR Knockout Results in a Deep Glycolytic and OXPHOS Reprogramming in Melanoma and Colon Carcinoma Cell Lines
Source: Cells. 2020 Jan 28;9(2):308. doi: 10.3390/cells9020308 (PMC7072355; doi:10.3390/cells9020308)
Supplement: Supplementary file 1 [file cells-09-00308-s001.pdf]

cells

MDPI

Article

uPAR Knockout Results in a Deep Glycolytic and OXPHOS Reprogramming in Melanoma and Colon Carcinoma Cell Lines

Alessio Biagioni [1,*], Anna Laurenzana [1], Anastasia Chillà [1], Mario Del Rosso [1], Elena Andreucci [1], Martina Poteti [1], Daniele Bani [2], Daniele Guasti [2], Gabriella Fibbi [1,†] and Francesca Margheri [1,†]

1 Department of Experimental and Clinical Biomedical Sciences, University of Florence, Viale G.B. Morgagni 50, 50134 Firenze, Italy; anna.laurenzana@unifi.it (A.L.); anastasia.chilla@unifi.it (A.C.); mario.delrosso@unifi.it (M.D.R.); e.andreucci@unifi.it (E.A.); martina.poteti@gmail.com (M.P.); gabriella.fibbi@unifi.it (G.F.); fmargheri@unifi.it (F.M.)

2 Department of Experimental and Clinical Medicine, University of Florence, Largo Brambilla 3, 50134 Firenze, Italy; daniele.bani@unifi.it (D.B.); daniele.guasti@unifi.it (D.G.)

* Correspondence: alessio.biagioni@unifi.it; Tel.: +39-055-275-1310

† These authors contributed equally to this paper.

Supplementary Materials

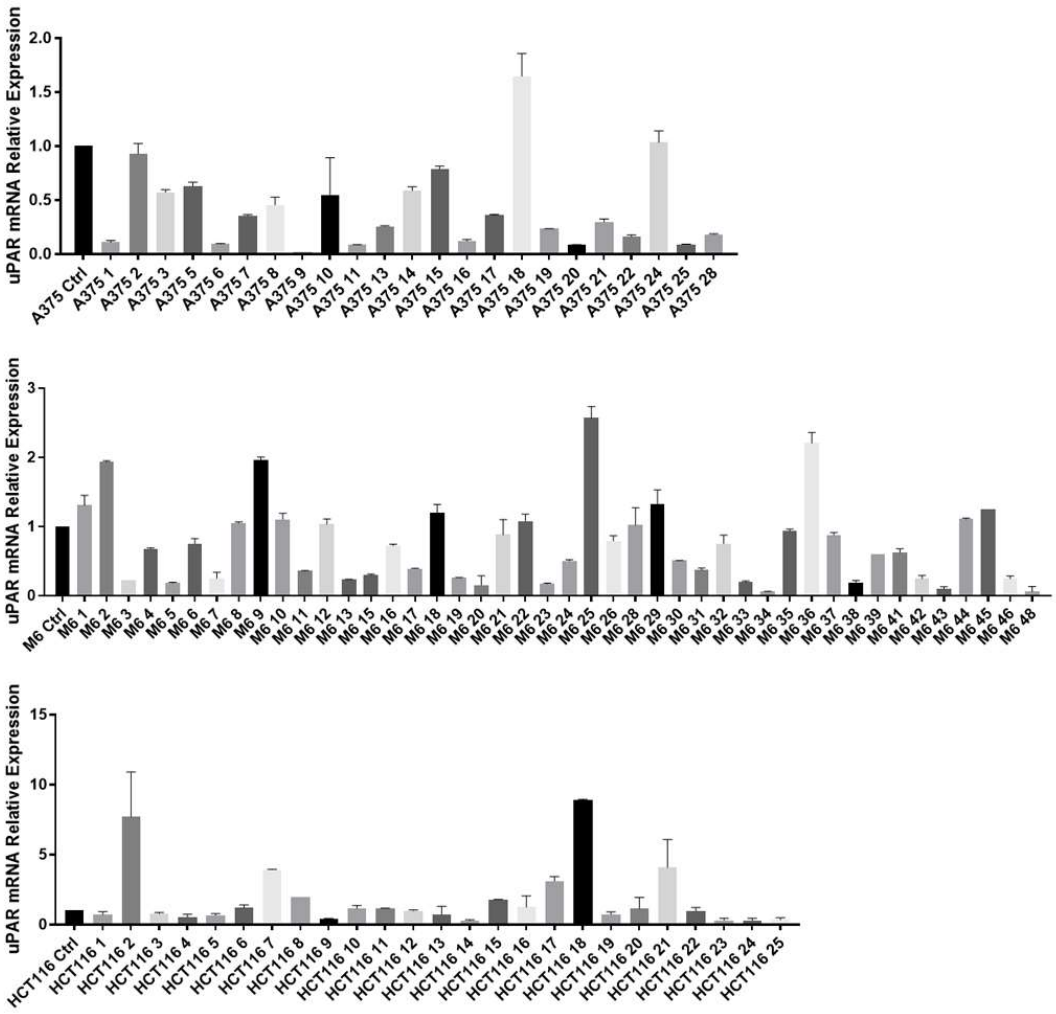

Figure S1. PLAUR expression screening after CRISPR transfection. Cells were transfected with PLAUR KO CRISPR plasmids, sorted for GFP marker, selected with 1μg/mL puromycin for 2–3 weeks and diluted limitingly in order to obtain single clones. Total RNA isolated from such clones was subjected to qPCR analysis of PLAUR expression. GAPDH and β2M were used as a loading control. Only the clones with a PLAUR expression under 0.15 were subsequently tested in Western Blot. The graphs shown above represent only the vital clones extracted and analyzed by the three multi 96-well plates, where the limiting dilution was performed, which gave successful results. The first one, the A375 1 clone, was demonstrated to be successfully KO and was named PL1 while clones M6 5 and HCT116 3, from the second and the third plates, were the ones called M6 A5 and HCT116 A3. Data are presented as mean ± SD.

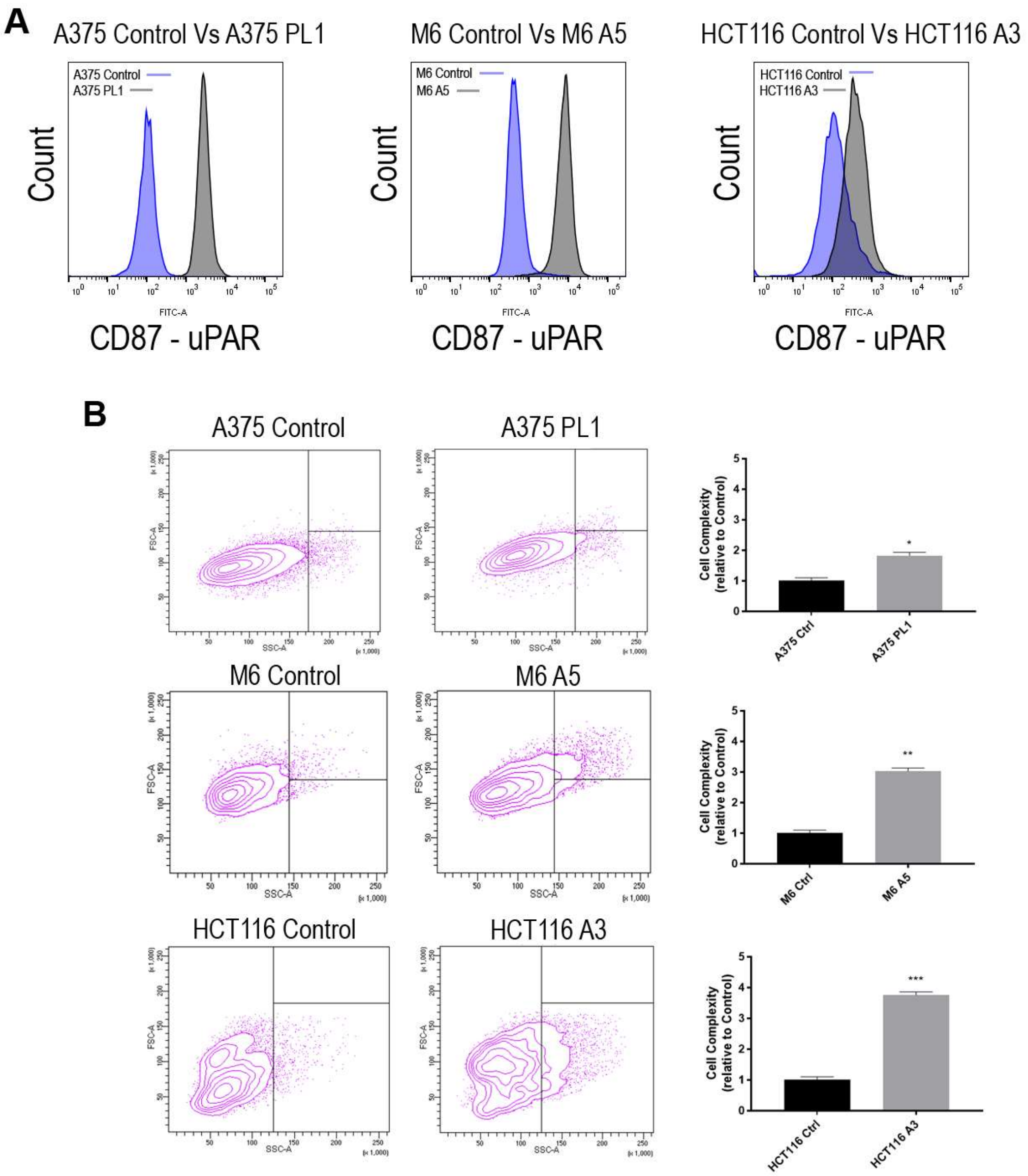

Figure S2. uPAR KO clones. (**A**) uPAR KO cells were tested for CD87 (uPAR) expression by FACS analysis. (**B**) Internal cell complexity was evaluated analyzing the SSC (Side Scatter) by FACS analysis. Data are presented as mean ± SD. * $p < 0.05$; ** $p < 0.001$; *** $p < 0.0001$.
